# Inflammation in Cerebral Amyloid Angiopathy‐Related Transient Focal Neurological Episodes

**DOI:** 10.1002/ana.27164

**Published:** 2025-01-08

**Authors:** Amina Sellimi, Larysa Panteleienko, Dermot Mallon, Simon Fandler‐Höfler, Rupert Oliver, Victoria Harvey, Michael S. Zandi, Gargi Banerjee, David J. Werring

**Affiliations:** ^1^ UCL Stroke Research Centre, Department of Brain Repair and Rehabilitation UCL Queen Square Institue of Neurology London UK; ^2^ Department of Neurology Cliniques Universitaires Saint‐Luc; Institute of Neuroscience, UCLouvain Brussels Belgium; ^3^ National Hospital for Neurology and Neurosurgery, Queen Square, University College London Hospitals NHS Foundation Trust London UK; ^4^ Department of Neurology Medical University of Graz Graz Austria; ^5^ Department of Neuroinflammation UCL Queen Square Institute of Neurology London UK; ^6^ MRC Prion Unit at UCL, Institute of Prion Diseases London UK

## Abstract

Transient focal neurological episodes (TFNE), often associated with convexity subarachnoid hemorrhage (cSAH), are common in cerebral amyloid angiopathy (CAA), but their pathophysiology remains incompletely understood. In six patients with unremitting TFNE, using high‐resolution post‐contrast magnetic resonance imaging and vessel wall imaging (VWI), we found various combinations of transient leptomeningeal, parenchymal and vessel wall enhancement; in 5 of 6 the enhancement included regions corresponding anatomically to symptoms. Three patients had resolution of TFNE and enhancement (2 with corticosteroid treatment, 1 without). Our observations suggest that inflammation might contribute to the pathophysiology of CAA‐related TFNE and cSAH, with potential wider relevance for the associated high risks of recurrent ICH in CAA more generally. ANN NEUROL 2025;97:475–482

Cerebral amyloid angiopathy (CAA) is a common, age‐related small vessel disease, defined histopathologically by the deposition of amyloid‐beta peptide in the walls of small cortical and leptomeningeal arteries, arterioles and capillaries in the brain.^1^ CAA is an established cause of stroke because of acute lobar intracerebral hemorrhage (ICH), and can be reliably diagnosed during life using magnetic resonance imaging (MRI) biomarkers.^2^

Transient focal neurological episodes (TFNE) are a common clinical presentation of CAA. Classically, these are brief (<30 minutes) stereotypical recurrent episodes of positive or negative symptoms (or, frequently, both)^3^ associated with hemorrhage from leptomeningeal vessels (ie, acute convexity subarachnoid hemorrhage [cSAH] and its chronic sequela, cortical superficial siderosis [cSS]).^4^ TFNE often occur in clusters (over days to weeks),^5^ and are associated with a high risk of subsequent intracranial bleeding (both cSAH and ICH).^6^, ^7^ However, the pathophysiology of TFNE remains incompletely understood.

In some cases, TFNE can be unremitting, with recurrent and frequent episodes—often with multiple attacks daily—over an extended duration. This is often distressing for patients and challenging for clinicians.

Post‐gadolinium contrast‐enhanced brain MRI and high resolution vessel wall imaging (VWI) provides a non‐invasive approach to evaluate blood–brain barrier (BBB) disruption and vasculopathic changes within individual small vessels.^8^, ^9^ Here, we describe 6 patients with unremitting CAA‐related TFNE in whom post‐gadolinium MRI identified abnormalities consistent with acute inflammation of small vessels, leptomeninges, and brain parenchyma.

## Methods

### Patients, Imaging, and Data Collection

This is a retrospective cases series of patients presenting with unremitted TFNE, which we defined as recurrent stereotyped episodes for at least 2 months at the time of assessment. All patients were assessed either at the specialist intracranial hemorrhage services at the National Hospital for Neurology and Neurosurgery, Queen Square, London, United Kingdom (n = 5), or the Department of Neurology, Medical University of Graz, Austria (n = 1), over a period of 12 months. Both institutions used the same imaging protocol including post‐gadolinium contrast‐enhanced brain MRI including high‐resolution VWI.

All patients met clinico‐radiological criteria for probable CAA,^2^ but not those for CAA‐related inflammation.^10^ Patients had the same standardized 3Tesla (T) MRI, including T2‐weighted, fluid attenuated inversion recovery, susceptibility‐weighted imaging, diffusion‐weighted imaging (DWI) sequences, as well as high‐resolution post‐contrast MRI including VWI, also called “black blood” imaging.^8^, ^9^

VWI was based on a post‐gadolinium volumetric single slab 3D turbo spin echo sequence using generalized autocalibrating partial parallel acquisition image acquisition acceleration and spectral attenuated inversion recovery fat suppression. A 180mm field of view was acquired with isotropic voxel size of 0.7mm, a repetition time of 900ms and an echo time of 9.7ms. VWI was acquired on either a Siemens Vida or Prisma_fit 3T MRI scanner.

The post‐contrast sequences were reviewed for leptomeningeal enhancement, parenchymal enhancement and vessel wall enhancement.

### Patient Consent

All patients gave informed consent for publication. No institutional review board approval was required as our investigations were part of our routine clinical practice.

## Results

The clinical and imaging characteristics of included patients are shown in Tables 1 and 2. The mean age was 70.4 years; 4 (67%) were female. All patients had evidence of cSS (83% disseminated [≥4 sulci] and 17% focal [≤3 sulci]). TFNE occurred at frequencies of between 3 per day and 2 per week. The median duration of TFNE at the time of imaging was 6 months (interquartile range [IQR] = 5 months).

**TABLE 1:** Summary of Clinical Features

	Case 1	Case 2	Case 3	Case 4	Case 5	Case 6
Age at presentation	74	70	84	59	70	66
Sex	F	F	F	M	F	M
CAA related‐TFNE characteristics						
Semiology	Right mouth/hand numbness; paresthesia, ~20 min	Slurred speech, right‐sided numbness, paresthesia (face, hand, leg), ~10–30 min	Left‐sided numbness (face, hand, arm), facial weakness, slurred speech ~10 min	Left‐sided paresthesia (hand, arm, face), ~10 min	Right numbness, paresthesia (tongue, face, hand, arm, thigh), ~4–5 min Left visual disturbance ~10–15 min	Left‐sided paresthesia (hand, arm, face), ≤1 min
Frequency	1–2/day	1–3/day	1–2/week	1–3/day	2–3/day	1–2/week
Interval between TFNE onset and first post‐contrast brain MRI	4 months	7 months	5 months	2 months	9 months	11 months
Interval between first and follow‐up brain MRI	3 months	6 weeks	–	2 months	7 months	5 months
Treatment for inflammation (after first post‐contrast brain MRI)	High dose oral corticosteroids	None	None	None	None	High dose corticosteroids
TFNE evolution during follow‐up	Resolution after 3 mo, reduced frequency after corticosteroids	Spontaneous resolution, persistent right leg paresthesia	Spontaneous resolution, occasional left hand altered sensation	Spontaneous resolution after 2 mo	Spontaneous resolution after 3 mo	Resolution 2 mo after corticosteroids

CAA = cerebral amyloid angiopathy; MRI = magnetic resonance imaging; TFNE = transient focal neurological episodes.

**TABLE 2:** Summary of Brain MRI Findings

	Case 1	Case 2	Case 3	Case 4	Case 5	Case 6
Findings of first post‐contrast MRI						
Markers of CAA						
Acute cSAH	Left perirolandic cSAH	−	−	Right central sulcus cSAH	−	Right inferior frontal sulcus cSAH
cSS	Disseminated	Focal	Disseminated	Disseminated	Disseminated	Disseminated
DWI‐hyperintense lesions	−	+	+	+	−	−
Post‐contrast MRI						
LME	Diffuse: left occipital and temporal lobe predominance	Focal: right occipital lobe, subcentral gyrus; left frontal parietal lobes; and superior cerebellar folia	−	Diffuse: bilateral parietal lobes predominance	Focal: left posterior cingulate sulcus, superior parietal lobule inferior right occipital lobe	Diffuse: right frontal sulcus predominance
VWE	−	−	−	−	Left ACA branch	Right M4 segment MCA
PE	−	Speckled: left frontal and parietal lobes	Speckled: right temporal and parietal lobes	−	Speckled: right superior frontal sulcus	Speckled: occipital lobes bilaterally and left frontal lobe
Anatomical‐ clinical correlation	+	+	−	+	+	+
Findings of follow‐up post‐contrast MRI						
Post‐contrast MRI						
DWI lesions	Left mesial temporal lobe	Right peritrigonal/lateral splenium	/	Right inferior occipital lobe	Superior frontal sulcus	Left frontal operculum and middle frontal gyrus
Enhancement	LME: reduced	No contrast imaging performed	/	No contrast imaging performed	LME: reduced	LME: reduced

ACA = anterior cerebral artery; CAA = cerebral amyloid angiopathy; CMB = cerebral microbleeds; cSAH = cortical subarachnoid hemorrhage; cSS = cortical superficial siderosis; (disseminated ≥4 sulci; focal, ≤3 sulci); DWI = diffusion‐weighted imaging; EPVS = enlarged perivascular spaces, LME = leptomeningeal enhancement; MCA = middle cerebral artery; MRI = magnetic resonance imaging; PE = parenchymal enhancement; VWE = vessel wall enhancement; WMH = white matter hyperintensities.

Three patients (cases 1, 4, and 6) had evidence of acute cSAH in an appropriate location corresponding to TFNE semiology at the time of presentation with TFNE, although episodes persisted despite resolution of acute hemorrhage. In the other patients, we did not find acute cSAH. None of the patients had typical neuroimaging features of CAA related inflammation (CAA‐ri).^11^

**FIGURE 1:**
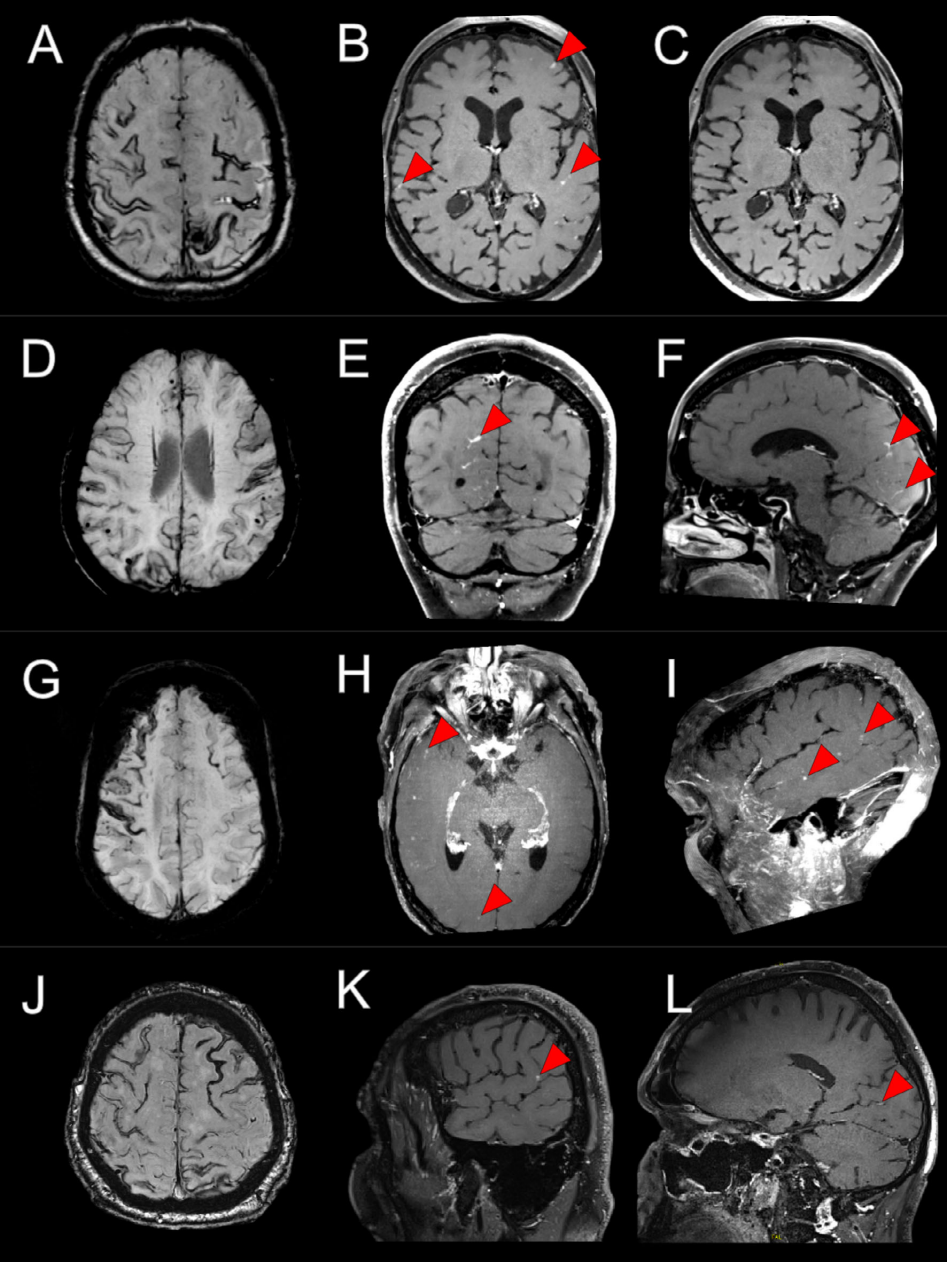
Each column contains magnetic resonance images for cases 1 to 4. In case 1, susceptibility‐weighted imaging showed extensive superficial siderosis (A). Vessel wall imaging (VWI) showed scattered foci of leptomeningeal enhancement over both cerebral hemispheres (B, *red arrows*). This enhancement resolved on follow‐up imaging performed 3 months later (C). In case 2, there was bilateral superficial siderosis and many lobar microhemorrhages (D). VWI showed thick leptomeningeal enhancement mainly in the right occipital lobe (E,F, *red arrows*). In case 3, there was superficial siderosis that was most apparent over the right frontal lobe (G). VWI maximum intensity projections showed punctate parenchymal enhancement in the right temporal and occipital lobes (H,I, *red arrows*). In case 4, there was extensive superficial siderosis (J) and lobar microhemorrhages (not shown). VWI showed punctate cortical and leptomeningeal enhancement in the right parietal lobe and within the right calcarine sulcus (K,L, *red arrows*). [Color figure can be viewed at www.annalsofneurology.org]

**FIGURE 2:**
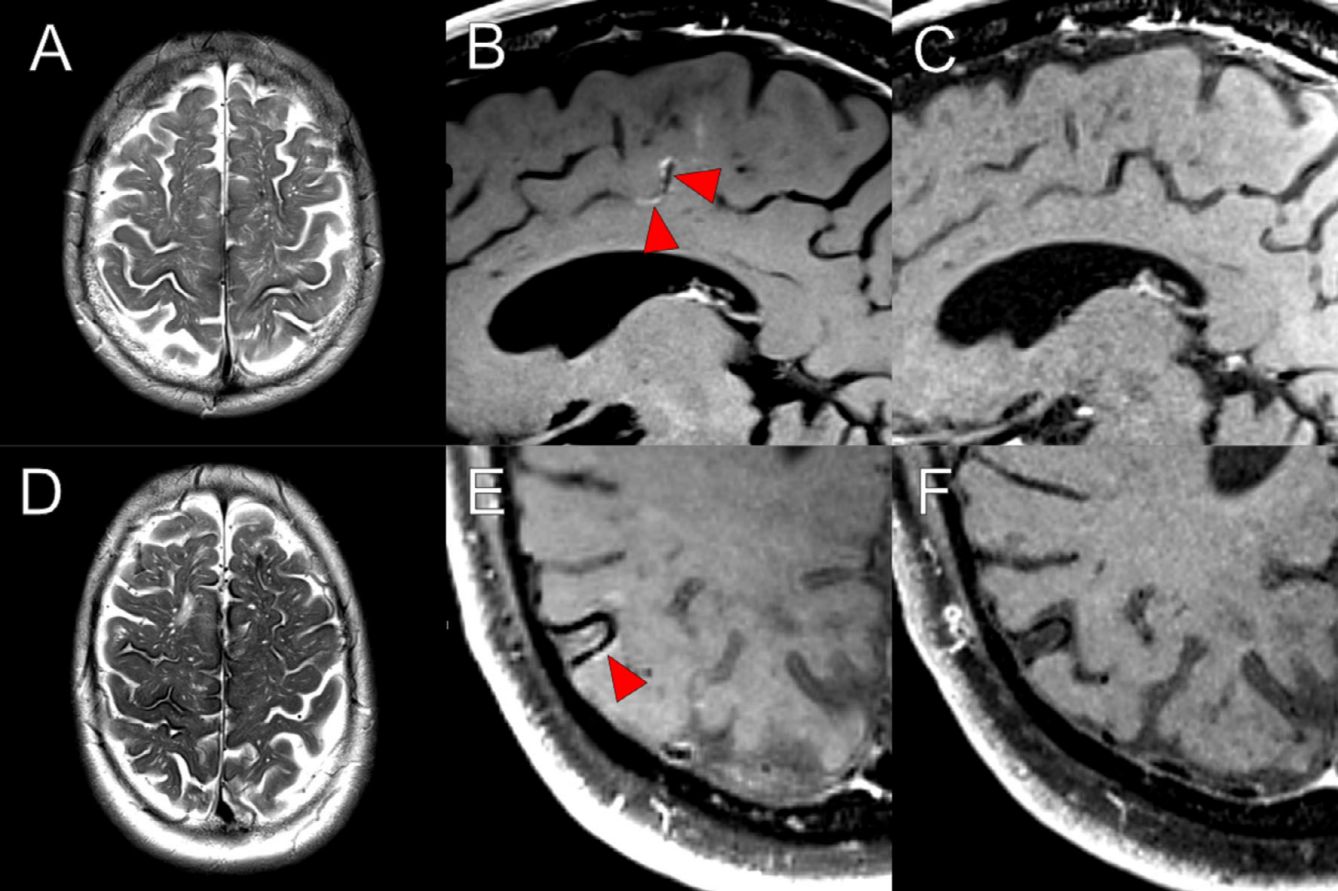
Each row contains magnetic resonance imaging for cases 5 and 6. In case 5, there was extensive superficial siderosis and many enlarged subcortical perivascular spaces (A). Vessel wall imaging showed irregular mural enhancement in a distal branch of the left anterior cerebral artery (*red arrows*, B), as well as a wider region of leptomeningeal enhancement, which resolved on follow‐up imaging 5 months later (C). In case 6, there was also extensive superficial siderosis and many enlarged perivascular spaces (D). A distal right middle cerebral artery branch showed a short segment of mural enhancement (E, *red arrow*) that resolved on follow‐up imaging 4 months later (F). [Color figure can be viewed at www.annalsofneurology.org]

High‐resolution post‐contrast MRI and VWI showed various combinations of abnormal enhancement (leptomeningeal, cortical parenchymal, or vessel wall enhancement) in all patients. Five (83%) had leptomeningeal enhancement (Fig 1), which was focal and non‐contiguous (n = 2) or diffuse and contiguous (n = 3). Two patients had vessel wall enhancement of small cortical vessels (Fig 2). Four patients had speckled cortical parenchymal enhancement. In 5 of 6 patients, there was some form of abnormal enhancement in an anatomical region corresponding to the TFNE semiology.

All patients had resolution of TFNE over a median follow‐up period of 3 months (IQR = 4.25 months). Three patients had follow‐up MRI with contrast. A further 2 had non‐contrast MRI only. Two patients (cases 1 and 6) had reduced contrast‐enhancement after high dose corticosteroids (methylprednisolone 500mg per day for 5 days), whereas in case 5 there was spontaneous improvement without treatment. We observed new small punctate DWI lesions in all 5 patients with follow‐up MRI in different brain areas from those previously affected by abnormal enhancement.

## Discussion

Using high‐resolution post‐contrast MRI with VWI, we describe various combinations of leptomeningeal, parenchymal, and small cortical arterial vessel wall enhancement, consistent with transient inflammation, in 6 patients with unremitting CAA‐related TFNE. In 5 of 6 cases, enhancement included regions correlated anatomically with TFNE symptoms, although the enhancement was generally rather diffuse, with a posterior (parieto‐occipital) predominance. These observations suggest that inflammation might play a role in the pathophysiology of unremitting CAA‐related TFNE, and, potentially, in CAA more generally.

The role of inflammation in sporadic age‐related CAA is poorly understood. One previous study found evidence of leakage of the meningeal vessels as a possible cause of recurrent intra‐sulcal bleeding,^7^ whereas a recent 7T MRI study of patients with sporadic and inherited (Dutch‐type) CAA described subarachnoid cerebrospinal fluid hyperintensities, potentially related to leptomeningeal inflammation, in close proximity to regions of cSS.^12^

Our findings might have relevance to the pathophysiological mechanism underlying TFNE, which affect approximately 15% of patients with CAA, and are important because of a high risk of future intracranial bleeding.^6^, ^13^ TFNE typically occur in clusters lasting days to weeks,^5^ and are thought to be triggered by blood breakdown products following cSAH within an eloquent cerebral sulcus leading to waves of cortical spreading depression.^4^ However, TFNE can occur in the absence of cSAH and in the presence of cSS alone, and the anatomical location of cSAH and/or cSS does not always correlate with TFNE semiology. Our observation of enhancement of the leptomeninges, small cortical arteries, and parenchyma, including in areas potentially anatomically relevant to the TFNE, suggests that inflammatory mechanisms might constitute another trigger for TFNE, with bleeding as a secondary consequence, or wothout evidence of bleeding, in some individuals. However, our observations cannot determine whether these inflammatory changes are a cause or consequence of bleeding or TFNE. For example, cortical spreading depression increases local oxygen and glucose consumption, which could disrupt the BBB with an upregulation of pro‐inflammatory cytokines, leading to inflammation.^14^

There are few previous studies of high‐resolution post‐contrast MRI and VWI in CAA. One previous study of 50 patients with CAA with acute focal neurologic deficits reported vessel wall enhancement in 29 of 50 (58%) patients,^15^ whereas another small series (n = 5) described vessel wall enhancement in 2 patients with histologically confirmed CAA without inflammation.^16^

These findings, together with our data, support the existence of inflammation in 'non‐inflammatory' CAA, and the use of VWI as a promising new technique for improving understanding of the pathophysiology of CAA and to explore inflammation and BBB leakage in vivo.

Inflammation might also have broader relevance for intracranial hemorrhage risk in sporadic CAA. Recent data on spatial–temporal clustering of ICH in CAA raise the possibility that a transient small vessel or leptomeningeal process, as observed in our study, could increase vascular fragility and result in ICH.^17^ Supporting this hypothesis, findings from a recent cross‐sectional neuropathological study in definite CAA suggest that BBB leakage and vessel contractility loss may precede arteriolar remodeling (ie, vessel wall fracturing and fibrinoid necrosis), and that perivascular inflammation around vessels with severe amyloid deposition might contribute to vessel rupture and intracranial hemorrhage.^18^

A recognized inflammatory subtype of CAA, often termed CAA‐related inflammation (CAA‐ri), is classically associated with a clinical syndrome characterized by encephalopathy, behavior change, headaches and seizures, and radiological features including asymmetrical, often migratory white matter changes, and hemorrhagic markers of CAA.^10^ CAA‐ri is characterized by transmural and/or perivascular inflammatory infiltrates^11^ and is often reversible with a good clinical and radiological response to immunosuppressive treatment (eg, corticosteroids).^10^

It is possible that prolonged TFNE might fall within the spectrum of CAA‐ri, although none of our patients meet current diagnostic criteria for CAA‐ri (eg, they did not have evidence of white matter edema).^10^ Furthermore, TFNE are not mentioned in current CAA‐ri diagnostic criteria and are not classically described as a clinical presentation of CAA‐ri.^11^ However, it may be possible that in CAA‐ri TFNE are masked by a more severe presentation, for example, encephalopathy or seizures, meaning that patients might not be able to report them.

Nevertheless, our observations could indicate an unrecognized role for various patterns of vascular, parenchymal or leptomeningeal inflammation in both sporadic CAA and inflammatory CAA, suggesting a possible inflammatory continuum with CAA‐ri as an extreme form at one end of this spectrum. Given that there are no current specific treatments to prevent ICH in sporadic CAA, the question of whether anti‐inflammatory or immunosuppressive treatments might help prevent intracranial hemorrhage merits further investigation.

In our report, 2 patients received treatment with high dose corticosteroids, with regression of abnormal enhancement and TFNE symptoms, consistent with a previous case report of a patient with CAA‐related TFNE associated with leptomeningeal arterial and sulcal venous vessel wall enhancement, which improved following corticosteroid treatment.^19^ However, another patient showed radiological improvement without treatment, so whether these observations indicate an effect of treatment or spontaneous improvement remains uncertain. Of note, we found new areas of enhancement, as well as new small DWI lesions in areas outside those previously affected by enhancement, suggesting dynamic and widespread disease processes. Further studies are needed to determine whether inflammation could be a trigger for the anatomically distirbuted small cortical and cortico‐subcortical DWI lesions commonly observed in sporadic CAA.^20^

Our observations therefore suggest that inflammation might, in some cases, contribute to the pathophysiology of CAA‐related TFNE and cSAH, with potential wider relevance for the associated high risks of recurrent ICH in CAA more generally.

Our study has limitations inherent to a small retrospective case series, so our observations need to be confirmed in larger prospective studies with appropriate control groups. Furthermore, VWI changes can also be observed in other intracranial vascular disease (eg, atherosclerotic plaques, vasculitis, and Moyamoya disease), but these diseases are typically not associated with parenchymal and leptomeningeal enhancement as observed in the current case series.^8^, ^9^

If confirmed, the described associations between prolonged and unremitted TFNE, cSAH, and the observed enhancement patterns might support the testing of anti‐inflammatory treatments as a preventive strategy in 'non‐inflammatory' CAA, which is currently a largely untreatable disease.

## Author Contributions

D.J.W. contributed to the conception and design of the study; all the authors contributed to the acquisition and analysis of data; A.S., G.B., and D.J.W. contributed to drafting the text; D.M. contributed to preparing the figures.

## Potential Conflicts of Interest

Nothing to report.

## Data Availability

The author has full access to the data used in this manuscript. The data are not publicly available because of privacy or ethical restrictions.
